# Suicide Attempt in a Poststroke Patient After Undergoing Deep Brain Stimulation: A Case Report

**DOI:** 10.7759/cureus.53520

**Published:** 2024-02-03

**Authors:** Christopher M Stevens, Amanda R Ragland, Sachin Nair, Juliana Fort

**Affiliations:** 1 Interventional Radiology, Louisiana State University Health Sciences Center, Shreveport, USA; 2 Medicine, Louisiana State University Health Sciences Center, Shreveport, USA; 3 Psychiatry, Louisiana State University Health Sciences Center, Shreveport, USA

**Keywords:** acute dystonia, stroke, depression, suicide, deep brain stimulation

## Abstract

Deep brain stimulation (DBS) is a type of therapy involving electrical stimulation of the brain and is primarily used to treat movement disorders. While perhaps beneficial, DBS has also been shown to have some potential major side effects, including increased risk for depression and suicide. In the present article, we report a case of a suicide attempt in a depressed patient two months after undergoing DBS for treatment of acute dystonia the patient had suffered from a prior ischemic stroke. This manuscript serves as a reminder of the negative ramifications that can be associated with DBS and why we should be cautious in providing DBS to patients who are either currently depressed or have a history of depression.

## Introduction

Deep brain stimulation (DBS), first approved in the 1990s for the treatment of movement disorders, is a type of therapy involving electrical stimulation of the brain [1]. Placement of the DBS apparatus consists of implanting electrodes into the brain that are then connected to a pacemaker-like device located in the chest wall via a subcutaneous wire [1]. DBS is approved by the Food and Drug Administration for the treatment of dystonia, Parkinson's Disease (PD), essential tremors, and refractory obsessive-compulsive disorder [2]. In 1999, Nuttin et al. published the first psychiatric use of DBS, leading to an increased interest in DBS as a potential therapeutic effect for certain conditions within the psychiatric community [3]. As of 2021, approximately 70 manuscripts a year discussing DBS in psychiatry are published [4].

While multiple studies have shown the potential therapeutic effects of DBS, studies have also suggested that DBS has serious potential side effects, including increased risk of depression and suicide [5-10]. A 2019 article by Accolla and Pollo even considered ongoing severe depression and suicidal ideation as absolute contraindications to one receiving DBS [5]; herein, we report a case of a suicide attempt in a depressed patient two months after undergoing DBS for treatment of acute dystonia (AD) that the patient had suffered from a prior ischemic stroke. This case highlights the potentially dangerous side effects of DBS while also demonstrating the importance of obtaining a thorough psychiatric history in patients receiving DBS. Additionally, to the authors’ best knowledge, this is the first documented case in which DBS performed on a poststroke patient potentially resulted in a suicide attempt, thus adding another intriguing detail to the report.

## Case presentation

A 72-year-old Caucasian male, with a past medical history of an ischemic stroke, Barrett’s esophagus, prostate carcinoma, essential hypertension, and mixed hyperlipidemia, a past psychiatric history of depression due to a medical condition, the condition being the ischemic stroke mentioned later in this text, and no family history of psychiatric disorders, presented to the emergency room (ER) via emergency medical services (EMS) for emergent evaluation of suspected overdose. Outpatient medications taken by the patient at this time included alprazolam 2 mg daily, atorvastatin 40 mg daily, clopidogrel 75 mg daily, dexlansoprazole 60 mg daily, and olmesartan 40 mg daily. Upon arrival, a Physician’s Emergency Certificate was executed, and a 0.5 mg naloxone injection was administered intravenously due to staff not being completely sure of what substance the patient consumed. The patient reported taking 15 x 1 mg alprazolam tablets, which the patient had been using for anxiety since November of 2021 after it was prescribed to him by his primary care physician, and stated that he was “ready to die.” According to the patient’s wife who was at the bedside, the patient took all 15 tablets approximately 16 hours before arriving at the ER but did not notify his wife until just before EMS was notified 16 hours later. The patient received supportive care, which consisted of routine check-ups by the nursing staff and psychiatry team to make sure the patient was not a danger to himself or anyone around him, before being transferred to an inpatient psychiatry facility. At his initial inpatient psychiatry evaluation the following morning, the patient was unable to remember parts of his visit to the ER from the night before and was unable to recall how he overdosed on his Xanax medication. When inquired about his depression, the patient became tearful and said he did not know how long he had been feeling depressed and felt like he was a failure to his wife and family for having the stroke. The patient was started on sertraline 25 mg once a day. 

The patient was in a psychiatric hospital for a total of nine days, in which the patient’s mood improved and there were no suicidal thoughts reported. During this time, the patient engaged with the treatment team on a routine basis and was cooperative in all the encounters. The patient was also compliant with his medication and endorsed no side effects. On the eighth day of the hospitalization, the patient’s sertraline was increased from 25 mg to 50 mg, with instructions for the patient to continue taking this dose after discharge. Upon discharge, the patient denied suicidal and homicidal ideations, auditory or visual hallucinations, and intent to abuse substances, and gave consent for his story to be shared and published. The patient was discharged home with transportation via his wife, and it was confirmed that the patient would have no access to guns or weapons. The patient is scheduled for monthly follow-up care at a psychiatric outpatient clinic; as of now, the patient is two months post-discharge and has not completed any of his follow-up visits at a psychiatry outpatient clinic. The patient had an appointment with a family medicine physician one month ago though and he reported doing well with no suicidal or homicidal ideations at that time.

**Figure 1:**
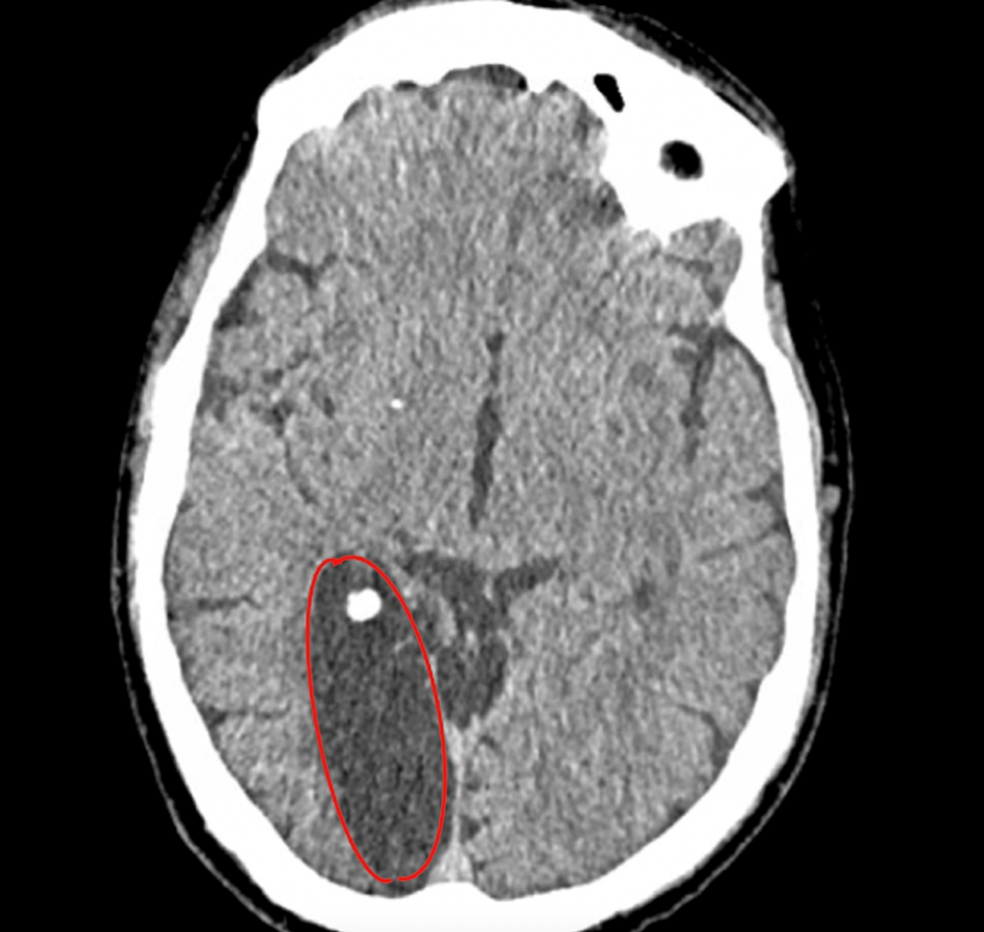
Non-contrast CT of the head showing the presence of encephalomalacia in the right posterior cerebral artery territory (red outline) due to a remote infarct

Approximately 19 months prior to this event, the patient was diagnosed with a chronic ischemic stroke of the right posterior cerebral artery that resulted in residual dystonia of the left upper extremity, left-sided numbness in both the upper and lower extremity, left-sided visual impairment, and aphasia (Figure 1).

At this time, the patient was discharged on dual antiplatelet therapy with aspirin and clopidogrel for 21 days followed by single platelet antiplatelet therapy with clopidogrel. After failed pharmacotherapy for the residual dystonia in his left upper extremity, which consisted of the patient trying valbenazine, clonazepam, diazepam, baclofen, gabapentin, levetiracetam, tizanidine, and olanzapine, the patient elected for a right-sided DBS of the ventral intermediate nucleus of the thalamus and internal globus pallidus approximately 17 months after his stroke (two months prior to his suicide attempt).

**Figure 2:**
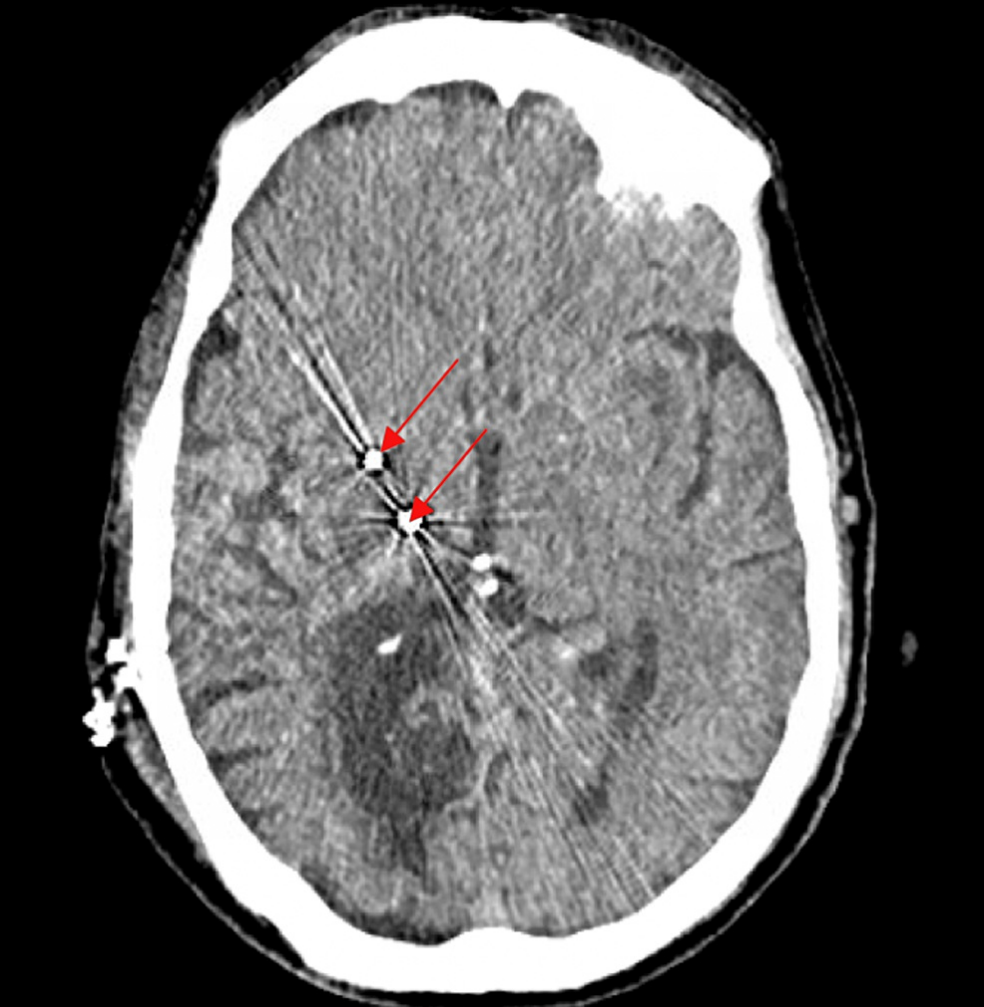
Non-contrast CT of the head showing the presence of right deep brain stimulator placement with electrode tips (red arrows) about the right globus pallidus internus and ventral intermediate nucleus of the thalamus

The patient visited psychiatry one month before the surgery and expressed feeling depressed and useless since his stroke but denied suicidal or homicidal ideations. Possible initiation of psychiatric medication was discussed at this visit, but the patient refused by saying, "I don't want to take anything for my mood. It is what it is and I just want to do the surgery." The patient still elected to have the surgery after being notified of the possible complications that could result from the surgery and the chance that the surgery could make his symptoms worse. The surgery was a success with no complications (Figure 2).

All AD medications were discontinued prior to surgery and none were reintroduced after surgery. Upon discharge, the patient continued to take atorvastatin 40 mg daily, olmesartan 40 mg daily, dexlansoprazole 60 mg daily, and hydrocodone-acetaminophen as needed for pain. Post-surgery follow-up visits at two, three, and four weeks saw the patient healing adequately from the surgery but no improvement in the patient’s dystonia. The patient expressed no complaints or signs of feeling depressed or suicidal during these postoperative visits.

## Discussion

AD, defined as sustained or interment involuntary muscle contractions that result in abnormal posturing or movement, is a potential complication patients can experience after having a stroke [11]. Although the pathophysiology of AD is not completely understood, dysfunction of the basal ganglia is believed to play an important role [12]. DBS is a neurosurgical procedure that involves the placement of a neurostimulator that sends electrical impulses to different parts of the brain through implanted electrodes, and it has been shown to be potentially beneficial for patients with AD [12,13]. While the pathophysiology explaining the therapeutic efficacy of DBS is still unclear, DBS seems to exert its therapeutic effects through multifactorial mechanisms, including synaptic plasticity, neuromodulator effects, and neuronal reorganization [14]; however, some potential side effects of DBS include enhanced depression, suicidal ideations, gait and speech disturbances, headaches, confusion, and difficulty concentrating [12,13]. In the present case, we present a patient who attempted suicide approximately two months after receiving DBS for poststroke AD. This manuscript serves as a reminder of the negative consequences that can be associated with DBS and why we need to monitor patients receiving DBS for possible onset or aggravation of depressive symptoms, including suicidal ideation or behavior. 

With stroke being the second leading cause of mortality worldwide, following heart disease, and many stroke patients suffering from disability related to neurological deficits [15], it is important for the scientific community to discover new ways to potentially help poststroke patients. DBS is starting to be introduced more into poststroke patients with movement disorders and other stroke-related complications due to recent research showing benefits from it [16-18]. While this new therapy can be beneficial, it is important for physicians to realize that there still is a risk with DBS in stroke patients, and the present case reminds us of this. A recent meta-analysis concluded that the prevalence of depression at any point and time after a stroke is roughly 27% [19], which is consistent with findings from other meta-analyses [20,21]. These findings, plus the fact that multiple studies show an association of DBS with depression and increased suicide risk as mentioned previously, means that poststroke patients receiving DBS are likely at an increased risk of experiencing a bad outcome with DBS; as such, physicians should be cautious when recommending DBS to these patients, and the patients should be notified of the increased risk of depression and suicidal ideation that sometimes accompanies DBS.

## Conclusions

Depression is a common occurrence in patients who have experienced a stroke, occurring in roughly 27% of these patients. Deep brain stimulation (DBS) has shown to be a potentially therapeutic procedure for movement disorders in poststroke patients, but increased risk of depression and suicide are complications of DBS that have been reported. In this report, we present a case in which DBS in a poststroke patient potentially propagated a suicide attempt, thus adding to the literature another example of the potential side effects DBS can have on a patient. This supports the idea that depression and suicide can be unexpected outcomes that need to be carefully monitored in patients receiving DBS.
